# The effects of extracellular contrast agent (Gadobutrol) on the precision and reproducibility of cardiovascular magnetic resonance feature tracking

**DOI:** 10.1186/s12968-016-0249-y

**Published:** 2016-05-21

**Authors:** Daniel L. R. Kuetting, Darius Dabir, Rami Homsi, Alois M. Sprinkart, Julian Luetkens, Hans H. Schild, Daniel K. Thomas

**Affiliations:** Department of Radiology, University of Bonn, Sigmund-Freud-Str.25, 53127 Bonn, Germany

**Keywords:** Feature tracking, Myocardial strain, Gadobutrol, Robustness, Cardiovascular magnetic resonance

## Abstract

**Background:**

Today feature tracking (FT) is considered to be a robust assessment tool in cardiovascular magnetic resonance (CMR) for strain assessment. The FT algorithm is dependent on a high contrast between blood pool and myocardium. Extracellular contrast agents decrease blood-myocardial contrast in SSFP images and thus might affect FT results. However, in a routine CMR scan, SSFP-cine images including short axis views are partly acquired after contrast agent injection. The aim of this study was to investigate the effect of extracellular contrast agent (Gadobutrol) (CA) on the precision and reproducibility of the feature tracking algorithm.

**Methods:**

A total of 40 patient volunteers (mean age 51.2 ± 19 years; mean LVEF 61 ± 9 %) were scanned in supine position on a clinical 1.5 T MR scanner (Philips Ingenia). SSFP-cine images in midventricular short axis view (SA) as well as horizontal long axis view (HLA) were acquired before and 10–15 min after injection of a double dose Gadobutrol. FT derived systolic circumferential and longitudinal strain parameters were then calculated for pre- and post-contrast images.

**Results:**

FT derived midventricular peak systolic circumferential strain (PSCS) (-24.8 ± 6.4 % vs. -20.4 ± 6.3 %), apical PSCS (-28.67 ± 6.5 % vs. -24.06 ± 8.5 %), basal PSCS (-24.42 % ± 6.5 vs. -20.68 ± 7.1 %), peak systolic longitudinal strain (-19.57 ± 3.3 % vs. -17.24 ± 4.1 %), midventricular epicardial PSCS (-9.84 ± 3.4 % vs. -8.13 ± 3.4 %) , midventricular PSCS-rate (-1.52 ± 0.4 vs. -1.28 ± 0.5) and peak diastolic circumferential strain rate (1.4 ± 0.5 vs. 1.05 ± 0.5) were significantly reduced after CA application. Post CA strain assessment showed higher intra- and interobserver variability. Pre-CA: intraobserver: mean 0.21, Limits of agreement (LoA) -2.8 and 3.2; interobserver: mean 0.64, LoA -2.8 and 4.1. Post-CA: intraobserver: mean -0.11, LoA -5.1 to 4.9; interobserver: mean 4.93 LoA 2.4 to 12.2.

**Conclusion:**

The FT algorithm is dependent on a high contrast between blood and myocardium. Post CA strain results are significantly lower and less reproducible than pre-CA strain results.

## Background

Myocardial strain imaging allows for objective quantification of cardiac contractility and is increasingly being recognized as a sensitive tool for analysis and diagnosis of various myocardial disorders [1, 2]. Furthermore strain analysis provides important prognostic information useful for predicting outcome of various ischemic and non-ischemic cardiomyopathies [3–5]. Although myocardial strain values were first derived from cardiovascular magnetic resonance (CMR) tagging (TAG) [6], echocardiography has been established as the clinical standard for the assessment of strain, as it is more widely available [7]. Feature tracking (FT) is a CMR post processing tool which allows for functional wall motion analysis in CMR cine steady-state free precession (SSFP) images and therefore opens the possibility of strain assessment in a standard clinical setting. The value of FT, but also its limitations, for assessment of regional and global systolic and diastolic strain have been demonstrated [8–10]. The CMR FT algorithm is based on an echocardiographic post processing tool, where strain assessment was first achieved without tagging. Voxels from the endocardial border are ascribed a certain number of features (e.g. brightness and dyshomogeneities of the tissue with respect to a 256-level gray scale) and then tracked from frame to frame [9], which enables a deduction of information about mechanical deformation. Extracellular contrast agents decrease the blood-myocardium contrast in SSFP images and thus might affect FT results. However in routine CMR, SSFP cine scans are frequently acquired after contrast agent injection to save time [11]. Thus the aim of this study was to investigate the effects of an extracellular contrast agent on the precision and reproducibility of the feature tracking algorithm.

## Methods

### Study population

40 patient volunteers (mean age 51.2 ± 19 years; mean LVEF 61 ± 9 %) were prospectively enrolled into the study. Blood pressure (BP) and heart rate (HR) were monitored during imaging. The study population included patients with suspected dilated cardiomyopathy, myocarditis and hypertrophic cardiomyopathy. None of the included patients had myocardial scar. Glomerular filtration rate (GFR) was controlled in all patients prior to contrast agent application, all patients had sufficient renal function (GFR: >40 ml/min). Written informed consent was obtained from all controls and patients. This study was approved by the institutional review board (Medical Ethics Committee - University of Bonn).

### CMR

CMR was performed on a clinical 1.5 T MR scanner (Intera, Philips Medical System, Best, the Netherlands) with a dedicated cardiac phased-array receiver coil. Scout images were acquired in axial, coronal and sagittal orientation. Cardiac functional imaging was performed using retrospectively gated SSFP sequences in the standard cardiac axes. For the assessment of ejection fraction, a minimum of 12 short axis slices (SA) were acquired per subject, with 30 phases reconstructed per slice. For assessment of strain additional retrospectively gated balanced-SSFP cine images in apical, midventricular and basal SA as well as horizontal long axis view (HLA) (each with 40 phases reconstructed per slice) were acquired before and 10–15 min after injection of a double dose contrast agent (CA) (Gadobutrol, Bayer HealthCare, concentration:1.0 molar(M)) as used for late enhancement imaging(0.2 mol/kg; average dose = 15.9 ± 3 ml). Typical scan parameters were: field of view 350 mm, slice thickness: 8 mm; NSA:1; TE 1.4 ms; TR 2.8 ms; Flip angle 50°; 40 phases per cardiac cycle.

### Strain assessment

CMR-FT strain analysis was performed using dedicated software (Diogenes; TomTec; Germany) which has been previously validated [4–6]. Circumferential strain *(Ɛcc)* values were derived from the apical-, mid- and basal-left-ventricular short axis slice. Longitudinal strain values were derived from a horizontal long axis slice. For strain analysis an initial endocardial contour is drawn in an end-diastolic phase with optimal contrast between blood and myocardium. The FT software then propagates the contour throughout the cardiac cycle. In case of faulty contour propagation the software allows editing throughout the cardiac cycle. Additionally, endocardial shortening was assessed in 20 subjects by calculating the percentage of end-diastolic to end-systolic shortening of the endocardial contour lengths in a midventricular SSFP image pre- and post-CA application.

### CMR strain indices

To investigate the effect of CA on FT strain assessment, established strain derived CMR indices (peak systolic circumferential strain (PSCS), peak systolic circumferential strain rate (PSCSR), peak diastolic circumferential strain rate (PDCSR)) were calculated for the midmyocardial slice in 40 subjects. Additionally, apical and basal PSCS systolic longitudinal strain (PSLS) as well as midventricular epicardial PSCS (EPSCS) were calculated for 20 patients. The peak strain rates were defined as the minimum respectively maximum values of the strain rate curve [7].

### Reproducibility of native and post-CA derived strain

Intra- and interobserver reproducibility of midmyocardial strain was investigated by two independent blinded observers in 20 randomly selected subjects. For assessment of intraobsever reproducibility an interval of two weeks was chosen between the first and second analysis.

### Contrast assessment

To investigate the reduction of the blood-myocardium contrast, a blood-myocardium contrast quotient was calculated in 20 subjects in a midventricular SSFP image pre and post CA application. Regions of interest (ROI) (minimum size: 80 mm^2^) were placed in the septum and the left ventricular lumen in an end-diastolic image. The signal average of the left ventricular lumen was divided by the signal average of the septum.

### Statistical analyses

Statistical analyses were performed using MedCalc (Mariakerke, Belgium). Results are expressed as mean ± SD. Normal distribution was tested with the D’Agostino-Pearson test. The Student t-test was employed for pre- and post-CA SSFP derived strain comparison if data were normally distributed, otherwise the Wilcoxon signed rank test was used. *P*-values of < 0.05 were considered statistically significant. Values for midventricular PSCS deducted pre- and post-CA were compared by the Pearson correlation coefficient for correlation and the Bland-Altman method [12] to assess agreement between the two observers and the two measurements. Increased variance post CA application was tested for using the coefficient of variation from duplicate measurements.

## Results

**Fig. 1:**
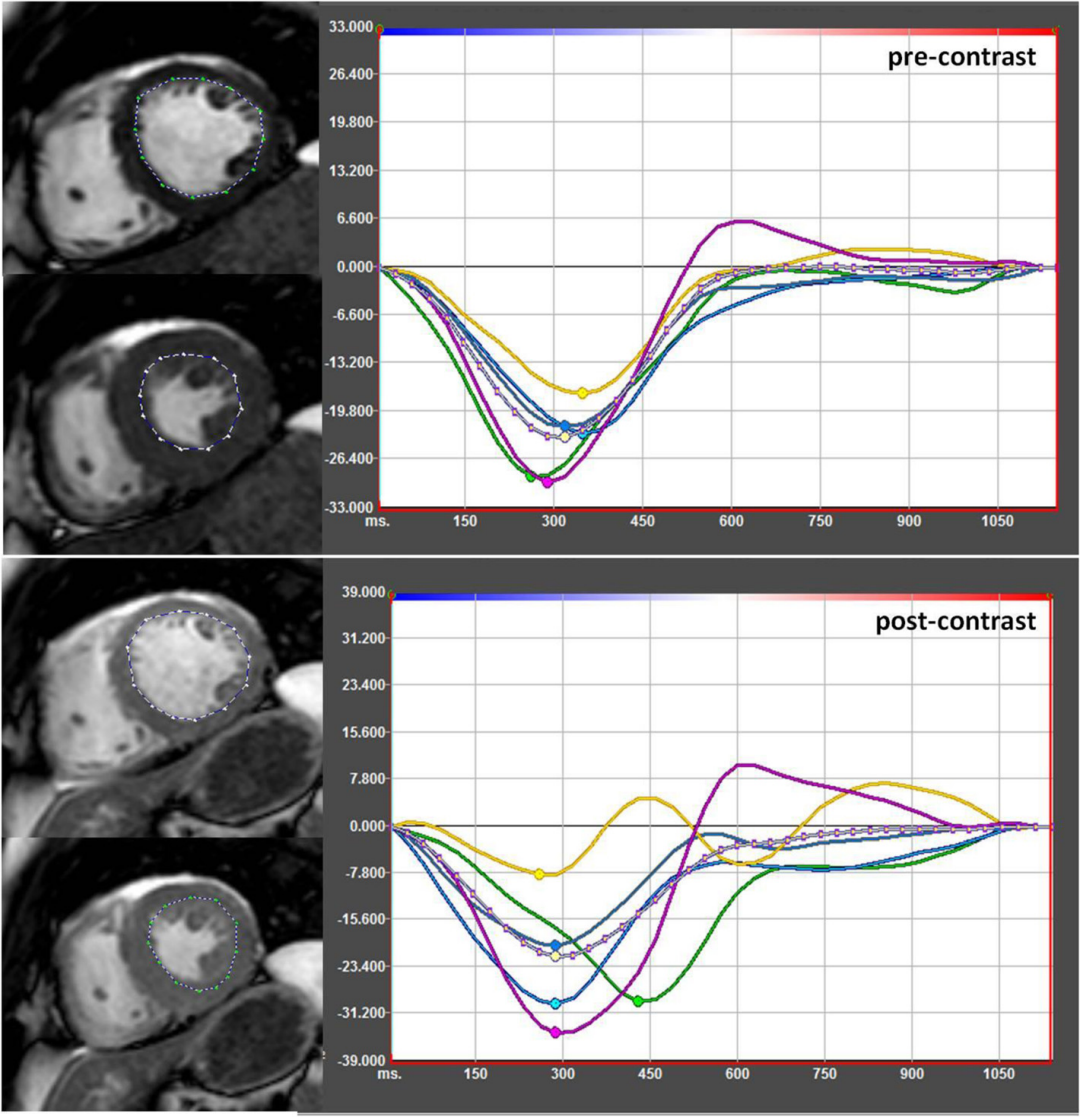
Example of FT strain assessment in SSFP images pre-contrast (upper images) and post-contrast (lower images) in the same subject. The dotted line represents the propagated contour in a end-diastolic- and end-systolic phase. The coloured curves in the graph represent the segments of the midventricular slice

The study protocol was completed by all participants. Table 1 summarizes pre- and post-CA results. Figure 1 demonstrates an example of typical *Ɛcc* curves derived by FT in a subject pre- and post-CA application. Mean HR (67.5 ± 13.6 BPM vs. 69.2 ± 13.9 BPM), mean BP ( 136.1 ± 18.2/72 ± 9 mm Hg vs. 138 ± 20.1/72.9 ± 8.2 mm Hg) as well as mean LVEF( 61 ± 9 % vs. 60 ± 9 %) did not differ significantly pre- and post-CA application. The blood-myocardium contrast quotient significantly decreased post CA application (4.03 ± 0.6 vs. 2.16 ± 0.3 *p* < 0.0001). No significant correlation was found between the degree of contrast reduction and the degree of strain reduction following CA application (*r* = 0.32). Assessment of endocardial shortening revealed no significant difference between pre-CA and post-CA images (27.97 ± 8.02 % vs. 27.63 ± 8.2 %).

**Table 1:** Baseline characteristics

Number of subjects	40
Age in years	51.2 ± 19
Female	40 %
Ejection Fraction	61 ± 9 %
LVEDV (ml)	137.3 ± 38.1
Average heart rate pre-CA (mm HG)	67.5 ± 13.6
Average heart rate post-CA (mm HG)	69.2 ± 13.9
Average blood pressure pre-CA (mm HG)	136.1 ± 18.2/ 72 ± 9
Average blood pressure post-CA (mm HG)	138 ± 20.1 / 72.9 ± 8.2
Average amount of injected CA (ml)	15.9 ± 3

Left ventricular enddiastolic volume (*LVEDV*), contrast agent (*CA*)

**Fig. 2:**
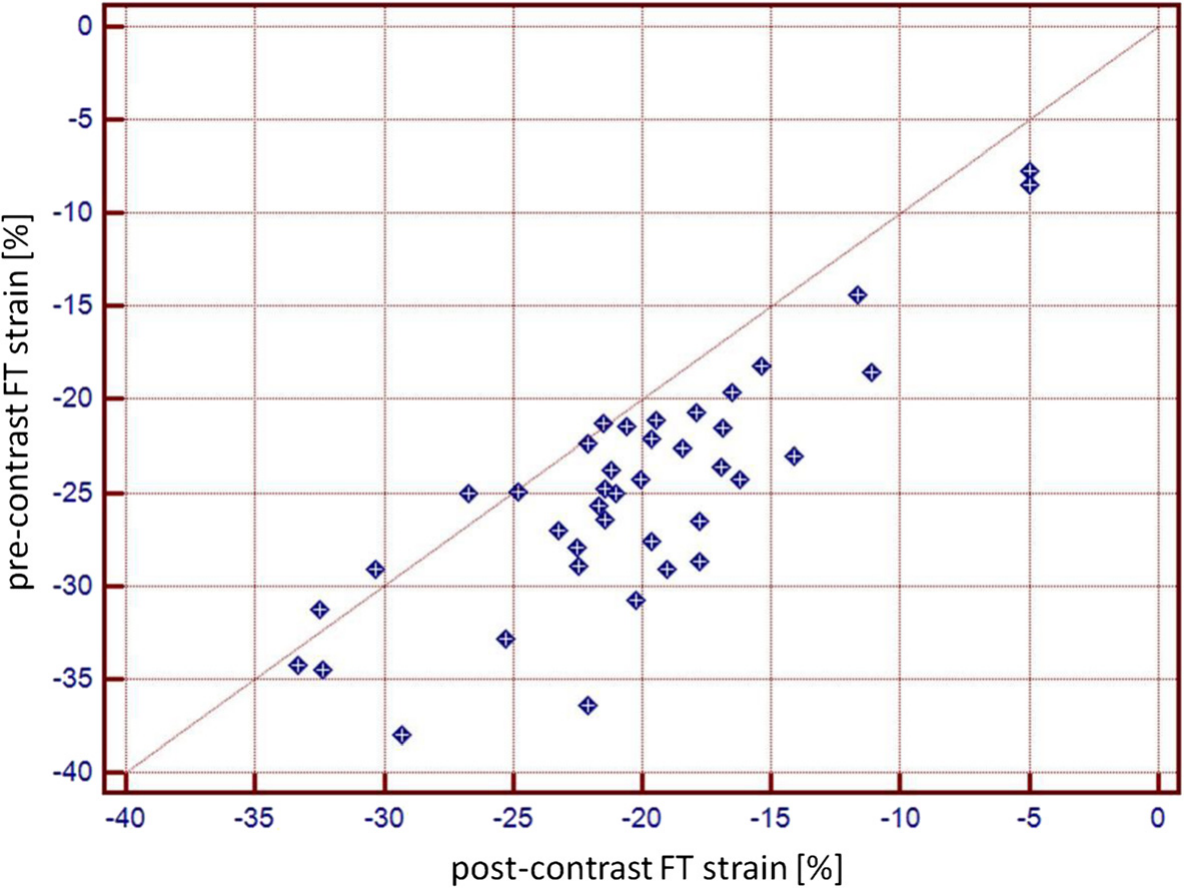
Pearson correlation coefficient between pre-contrast and post-contrast derived strain (*r* = 0.81)

10–15 min after CA application FT derived midventricular PSCS (-24.8 ± 6.4 % vs. -20.4 ± 6.3 %), apical PSCS (-28.67 ± 6.5 % vs. -24.06 ± 8.5 %), basal PSCS (-24.42 % ± 6.5 vs. -20.68 ± 7.1 %), PSLS (-19.57 ± 3.3 % vs. -17.24 ± 4.1 %) and midventricular EPSCS (-9.84 ± 3.4 % vs. -8.13 ± 3.4 %) were significantly reduced in comparison to baseline strain analysis. Furthermore, midventricular PSCSR(-1.52 ± 0.4 vs. -1.28 ± 0.5) and PDCSR(1.4 ± 0.5 vs. 1.05 ± 0.5) were also significantly reduced after CA application (Tables 2 and 3). Correlation for pre- and post-CA derived midventricular PSCS was *r* = 0.81 (Fig. 2).

**Table 2:** Results for pre- and post- contrast midventricular circumferential strain

	native (*n* = 40)	post-CA (*n* = 40)	*p*
Mid PSCS (%)	24.8 ± 6.4	20.4 ± 6.3	<0.005
Mid PDCSR (s^−1^)	1.4 ± 0.5	1.05 ± 0.5	<0.005
Mid PSCSR (s^−1^)	−1.52 ± 0.4	−1.28 ± 0.5	<0.05

Midventricular (*Mid*), peak systolic circumferential strain (PSCS), peak diastolic circumferential strain rate (*PDCSR*), peak systolic circumferential strain rate (*PSCSR*)

**Table 3:** Results for pre- and post- contrast apical, midventricular and basal circumferential strain as well as longitudinal strain

	native (*n* = 20)	post-CA(*n* = 20)	*p*
apical PSCS (%)	−28.67 ± 6.5	−24.06 ± 8.5	<0.005
basal PSCS (%)	−24.42 ± 6.5	−20.68 ± 7.1	<0.005
HLA PSLS (%)	−19.57 ± 3.3	−17.24 ± 4.1	<0.05
midventicular EPSCS (%)	−9.84 ± 3.4	−8.13 ± 3.4	<0.05

Horizontal long axis (*HLA*), peak systolic circumferential strain (*PSCS*), peak systolic longitudinal strain (*PSLS*), epicardial peak systolic circumferential strain (*EPSCS*)

**Fig. 3:**
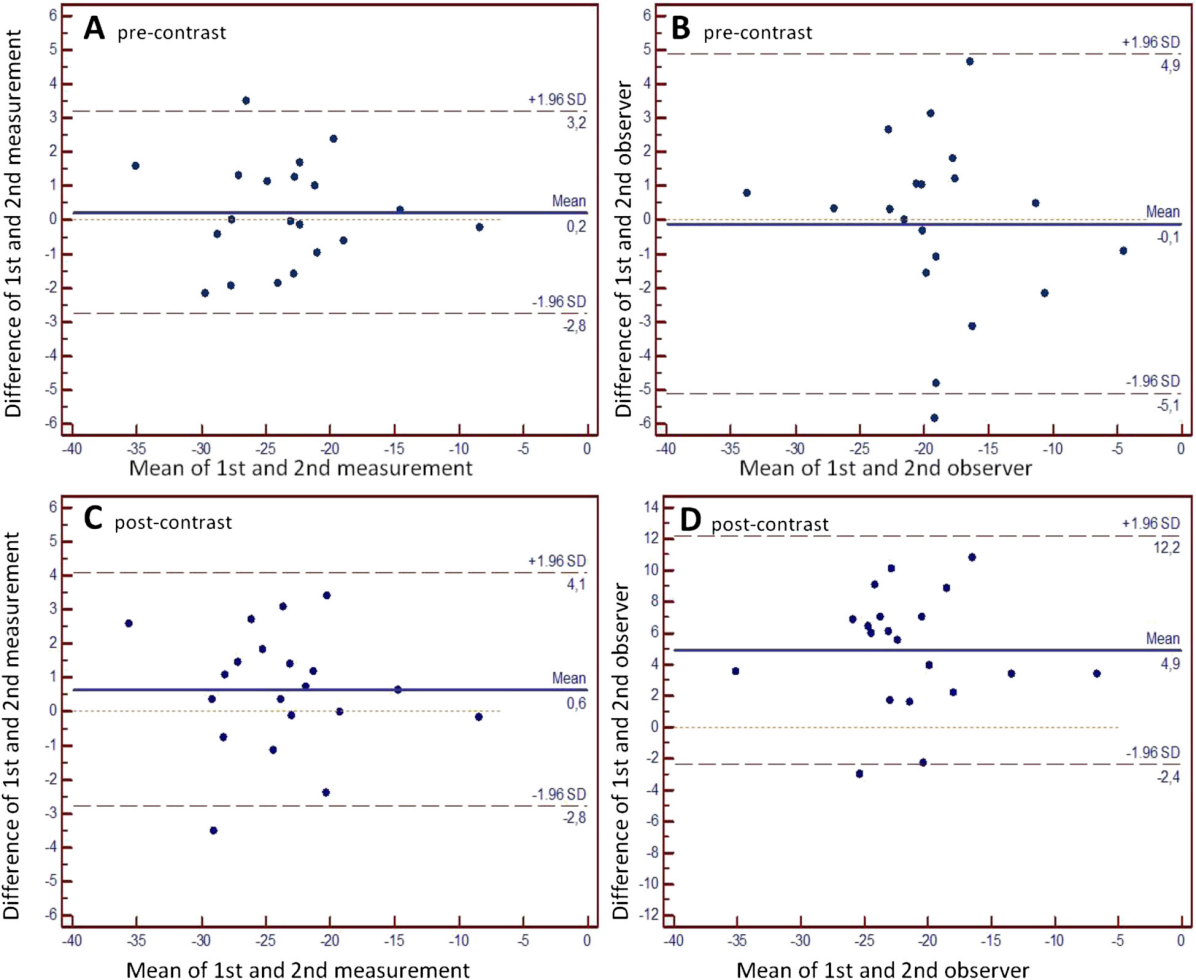
Panel **a** Bland Altman plot of intraobserver variability for pre-contrast midventricular FT derived peak circumferential systolic strain (PSCS). Panel **b** Bland Altman plot of interobserver variability for pre-contrast midventricular FT PSCS. Panel **c** Bland Altman plot of intraobserver variability for post-contrast midventricular FT derived PSCS. Panel **d** Bland Altman plot of interobserver variability for post-contrast midventricular FT PSCS

Bland Altman analysis of pre-CA intra-observer reproducibility yielded a better agreement (0.21 ± 1.5 with limits of agreement between -2.8 and 3.2) than post-CA intra-observer reproducibility (-0.11 ± 2.5 with limits of agreement between- -5.1 to 4.9). Correspondingly interobserver variability (0.64 ± 1.75 with limits of agreement between -2.8 and 4.1 vs. 4.93 ± 3.7 s with limits of agreement between- -2.4 to 12.2) was superior pre-CA (Fig. 3). Correlation coefficients for pre-CA derived strain were excellent (Intraobserver: *r* = 0.97) (Interobserver: *r* = 0.95) and superior in comparison to post-CA derived strain (Intraobserver: *r* = 0.91) (Interobserver: *r* = 0.91). The intra- and interobserver coefficients of variation were 4.5 and 5.4 %, for pre-CA strain assessment, 22.8 % and 20.9 % for post-CA strain assessment respectively.

## Discussion

The current gold standard for myocardial strain imaging in CMR remains tagging [13–15]. Several techniques such as displacement encoding with stimulated echoes(DENSE) [16], complementary SPAMM (CSPAMM) [17], harmonic phase (HARP) [18] and strain encoding (SENC) [19] have been developed to expedite and optimize the tagging derived strain analysis. These techniques are based on the creation of a pattern of magnetization saturation, from which movement throughout the RR cycle can then be quantified [20]. In comparison, the FT algorithm does not deduct strain information from a created strain pattern. The advantage of FT is that additional complex imaging and postprocessing is no longer necessary for strain analysis. However, the FT algorithm is highly dependent on a high contrast between blood and myocardium [15] and in comparison to tagging techniques FT has been found to be less robust [8, 10]. For comparison purposes, the identical sequence parameters, including identical flip angles, were used for pre- and post-CA imaging. In order to have identical sequences, the flip angle was not adapted for post CA imaging. In this study, reducing contrast by acquiring SSFP images after CA injection delivered significantly different strain-results as well as inferior reproducibility in comparison to strain analysis based on pre-CA images. Circumferential apical-, midventricular- and basal- PSCS, PSLS as well as midventricular systolic and diastolic strain rates were significantly reduced when derived from post-CA SSFP images, while neither heart rate nor blood pressure varied. Post CA midventricular EPSCS showed less severe reduction following CA application in comparison to midventricular PSCS, a possible explanation may be that the epicardial fat-myocardial interface is less affected by CA administration than the endocardium blood pool interface.

The CA Gadobutrol, is not known to cause changes in heart rate, blood pressure or to affect the cardiac conduction system [21]. An average of 15.9 ± 3 ml of CA followed by 25 ml of saline flush were injected per patient for late enhancement imaging. This small volume is not likely to have affected preload or contractility, furthermore increased preload will typically increase cardiac contractility and strain, contrary to the reduction that was found. Although to date the different effects of the various gadolinium based contrast agents on the blood pool-myocardium contrast have not been evaluated, it is to be expected that in case of application of gadopentetate dimeglumine (0.5 M) the effect on contrast will be similar or even worse compared to those of Gadobutrol (1 M), especially as gadopentetate dimeglumine exhibits a weak, transient interaction with serum albumin possibly decreasing the blood pool-myocardium contrast even more [22]. Previous studies assessing FT have found mixed results for reproducibility, with several studies reporting considerable intra- and interobserver as well as interstudy variability especially for regional, as well as apical and basal derived FT derived strain [8, 10, 23, 24], underlining its restricted robustness in comparison to tissue tagging. In the current study pre-CA reproducibility was comparable to results from previous studies [9, 23], while post-CA derived strain reproducibility showed inferior results. Post-CA FT derived strain was reduced in comparison to pre-CA FT derived strain in almost all cases, with corresponding results for intra- and interobserver repeated analysis indicating that contrast reduction leads to an underestimation of strain. Increased intra- and interobserver variability as well as an increased coefficient of variation post CA application however indicate that the error caused by contrast reduction is not truly systematic, as a systematic error should not influence reproducibility. Furthermore the degree of strain reduction did not correlate with the degree of contrast reduction further indicating that CA application leads to an unsystematic underestimation of FT derived strain. The FT algorithm calculates strain based on the detection and tracking of contrasts and dyshomgeneities of a cluster of voxels from frame to frame throughout the RR-cycle. When the initially characterized cluster cannot be re-detected in the following phase, apparently a different cluster of voxels is tracked leading to a false result. As the FT software does not provide information on tracking quality, it is essential to employ FT only in cases with good image quality and myocardium-blood contrast. It is important to note that several cardiac diseases, which can potentially be detected by CMR strain analysis, are often characterized by only mild systolic or diastolic strain impairment [25, 26]. Thus, the highest possible precision is demanded from clinically employed strain assessment tools. A higher variability of strain measurements, as can be found when deducting strain from post CA images, means that mild systolic or diastolic dysfunction could either be overlooked or even falsely diagnosed.

The following limitations apply to this study: Both post-contrast and pre-contrast SSFP were acquired with the same imaging parameters. Thoughtful adaptation of pulse sequence parameters (e.g.flip angle) may increase blood myocardium contrast post CA injection. However, in this study pulse sequences were chosen to reflect the clinical routine. As discussed above endocardial shortening and ejection fraction were used as surrogate markers to rule out an effect of Gadobutrol on contractility, however, the gold standard for strain imaging -namely myocardial tissue tagging- was not employed.

## Conclusion

FT has become an established tool for rapid SSFP based strain analysis, however it is dependent on a high contrast to allow for precise voxel tracking from frame to frame. Assessing strain from post CA SSFP cine images, which leads to a reduction in the myocardium-blood contrast, leads to an unsystematic underestimation of strain with increased intra- and interobserver variability. The strain based evaluation of many ischemic- and non-ischemic- cardiomyopathies demands the highest possible precision and robustness, therefore FT based strain analysis should only be performed on pre CA SSFP cine images.

## Abbreviations

CA: contrast agent
LVEF: left ventricular ejection fraction
SSFP: steady state free precession
CMR: cardiovascular magnetic resonance
SA: short axis
HLA: horizontal long axis
FT: feature tracking
PSCS: peak systolic circumferential strain
PSLS: peak systolic longitudinal strain
PSCSR: peak systolic circumferential strain rate
PDCSR: peak diastolic circumferential strain rate
BP: blood pressure
HR: heart rate
*Ɛcc*: Circumferential strain
DENSE: displacement encoding with stimulated echoes
CSPAMM: complementary SPAMM
HARP: harmonic phase
SENC: strain encoding
